# Translating pharmacogenomics into clinical decisions: do not let the perfect be the enemy of the good

**DOI:** 10.1186/s40246-019-0229-z

**Published:** 2019-08-27

**Authors:** Kristi Krebs, Lili Milani

**Affiliations:** 1 Estonian Genome Center, Institute of Genomics, University of Tartu, Tartu, Estonia; 2 Institute of Molecular and Cell Biology, University of Tartu, Tartu, Estonia

**Keywords:** Pharmacogenetics, Pharmacogenomics, PGx, Implementation of pharmacogenetics, Translation into the clinic, Clinical decision support

## Abstract

The field of pharmacogenomics (PGx) is gradually shifting from the reactive testing of single genes toward the proactive testing of multiple genes to improve treatment outcomes, reduce adverse events, and decrease the burden of unnecessary costs for healthcare systems. Despite the progress in the field of pharmacogenomics, its implementation into routine care has been slow due to several barriers. However, in recent years, the number of studies on the implementation of PGx has increased, all providing a wealth of knowledge on different solutions for overcoming the obstacles that have been emphasized over the past years. This review focuses on some of the challenges faced by these initiatives, the solutions and different approaches for testing that they suggest, and the evidence that they provide regarding the benefits of preemptive PGx testing.

## Background

The promise of pharmacogenomics (PGx) is that the use of an individual’s’ genetic information would help to predict drug response and further guide optimal drug and dose selection to enable safer, more effective, and cost-effective treatment [1]. Research in PGx variability goes back several decades and, within the last 10 years, more and more initiatives to implement PGx associations in the clinic have finally started to emerge. Many health institutions have implemented pharmacogenetics reactively, on a gene by gene basis, ordering a test when there is a need to prescribe a high-risk drug, to ensure that the optimal treatment is selected. However, reactive implementation is expensive and has a slow turnaround time that might even turn out to be irrelevant when a rapid drug prescription is necessary. As technology advances, it is becoming increasingly more recognized that PGx testing results of the broad screening of multiple pharmacogenes as well as recommendations for dosing need to be available preemptively in electronic health records (EHR) and drug prescription systems [2]. The preemptive translation of PGx discoveries remains a challenge, but implementation efforts have brought and will bring more informed knowledge to constantly improve solutions.

**Fig. 1:**
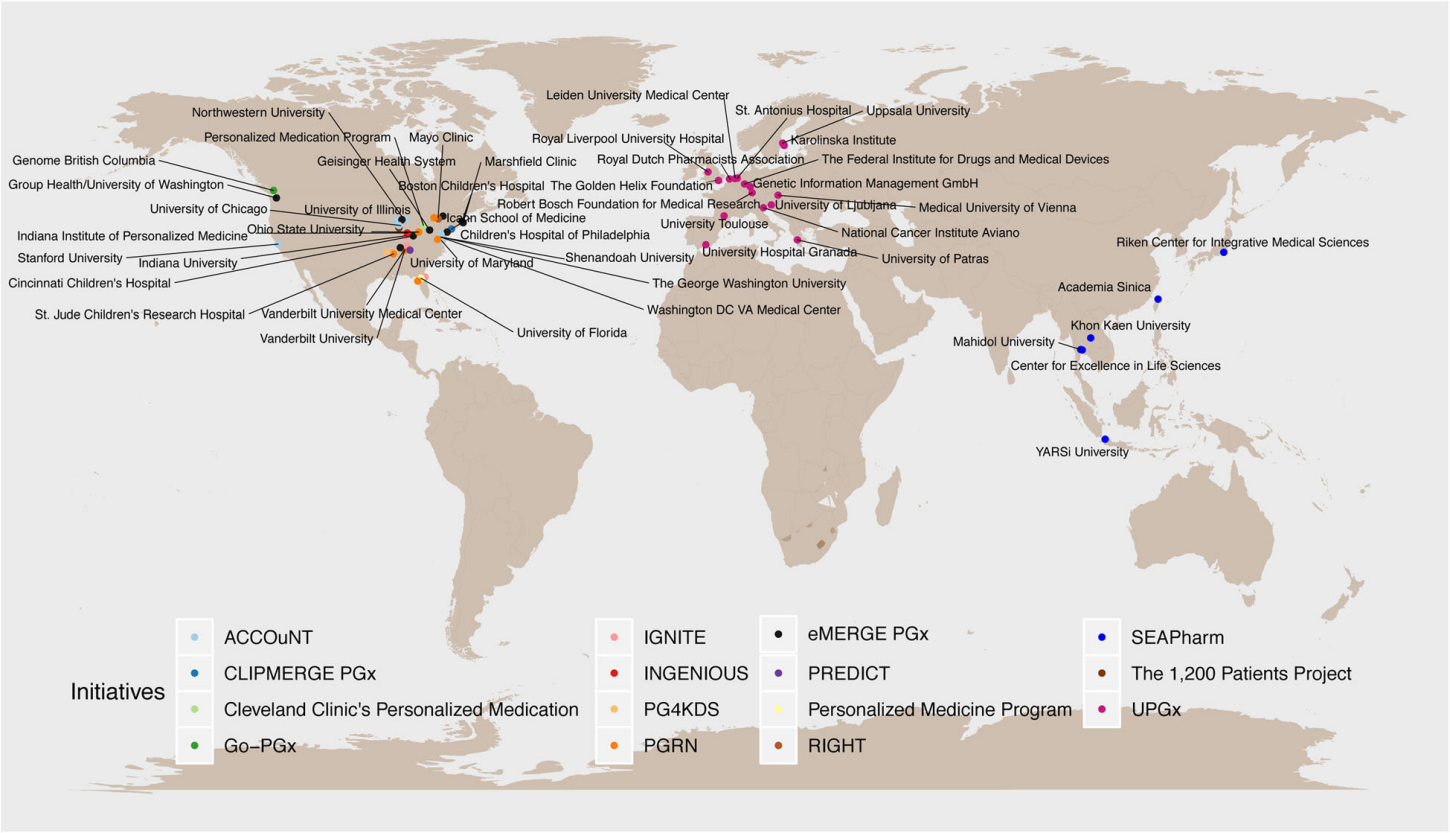
Current pharmacogenetic implementation initiatives. Colored points indicate different programs and consortia established for collaborative PGx implementation studies (details in Table 1)

Currently, various reported, ongoing initiatives of PGx implementation have been launched in the United States (US), Europe, and Asia (Fig. 1, Table 1) [2, 17–19]. In the US, 27 different institutions are involved in programs that are implementing pharmacogenomics, some of which have been going on for over 10 years. In 2007, a large network of several consortia was initiated with the establishment of the Electronic Medical Records and Genomics (eMERGE) Network. They later started a study named eMERGE-PGx together with the Pharmacogenomics Research Network (PGRN), with the aim of testing genetic variation in 82 pharmacogenes through targeted sequencing [5]. Their February 2015 data release included 5639 samples sequenced from nine eMERGE sites [20]. Since September 2010, with the Vanderbilt Pharmacogenomic Resource for Enhanced Decisions in Care and Treatment program (PREDICT) [13], more than 10,000 patients have undergone preemptive, panel-based pharmacogenomic testing [21]. In 2011, PGRN also started the Translational Pharmacogenetics Program to assess PGx implementation in routine care further by identifying barriers and developing solutions [12, 22]. When moving from the US to Europe, the EU-funded Ubiquitous Pharmacogenomics (U-PGx) Consortium was formed in 2017. It is a network of European experts that aims to assess and provide evidence of the clinical utility of a panel of PGx-markers in a multidrug, multigene, multicenter, multi-ethnic approach. Across seven European countries, a panel of clinically relevant PGx-markers will be preemptively genotyped and the effect on patient outcomes will be investigated, conducting a controlled clinical study of PREemptive Pharmacogenomic testing for prevention of Adverse drug REactions (PREPARE) [17]. Moving further to Asia, the South East Asian Pharmacogenomics Research Network (SEAPharm) program was established by five Asian countries (Korea, Indonesia, Malaysia, Taiwan, and Thailand) to conduct trial studies of adverse drug effects and develop guidelines adapted to Asian populations, which could guide drug use and prove useful in disease prediction/diagnosis [15, 23].

**Table 1:** An overview of some pharmacogenetic implementation initiatives and institutes involved

Project	Goals	References
ACCOuNT (African American Pharmacogenomic Consortium Network)	Move studies of African American pharmacogenomics from discovery to implementation; guidance for developing genomic prescribing system; developing recommendations that consider ethnic background	[3]
CLIPMERGE PGx	Develop best-practice infrastructure for PGx implementation; real-time clinical decision support (CDS); the utility of genomic information in optimizing medication efficacy and safety	[4]
eMERGE-PGx (Electronic Medical Records and Genomics)	Integration of clinically validated genotypes to EHR and CDS; measuring outcomes and cost-effectiveness; repository of variants of unknown significance for the expansion of PGx understanding	[5]
Go-PGx (Genomic and outcomes database for pharmacogenomics and implementation studies)	Genomics-based precision health strategies to reduce the most common and serious ADRs; incorporate tests into clinical practice; study barriers; economic implications of testing in clinical practice	[6]
IGNITE (Implementing GeNomics In practice)	Evaluate the feasibility of incorporating genomic information into clinical care; define, share and disseminate the best practices of implementation; contribute to the evidence base of outcomes of the use of genomic information in clinical practice	[7]
INGENIOUS (INdiana GENomics Implementation: an Opportunity for the UnderServed)	Evaluate adverse event incidence and annual healthcare cost, integration of results through the EHR and clinical decision support system	[8]
Personalized Medication Program	Incorporate genetics into the medical decision-making process; develop the implementation tools needed to incorporate pharmacogenomics into the clinical workflow; implement clinical decision support system to guide test ordering and PGx recommendations at the point of care	[9]
Personalized Medicine Program	Expand and evaluate the clinical implementation of PGx information; identification of the common challenges; educational programs targeted at health science students	[10]
PG4KDS	Establish processes for using PGx tests in the EHR to pre-emptively guide prescription; develop interruptive CDS alerts; educational efforts for both patients and clinicians	[11]
PGRN (Pharmacogenomics Research Network) translation PGx program	Assessment of the implementation of routine evidence-based PGx testing; templates for reporting results with CDS; educational materials for clinicians; gene–drug pair clinical guidelines	[12]
PREDICT (The Pharmacogenomics Resource for Enhanced Decisions in Care and Treatment)	Develop infrastructure and a framework for incorporating PGx results into the EHR and making these available to clinicians at the time of prescription	[13]
RIGHT (Right drug, right dose, right time)	Develop best practice protocol for implementing genetic sequence data; point-of-care CDS	[14]
SEAPharm (South East Asian Pharmacogenomics Research Network)	Studies of adverse drug effects and developing guidelines adapted for the Asian population	[15]
The 1200 Patients Project	Establish a model system for eliminating practical barriers to implementing PGx; Interactive consultation portal for physicians; Clinical relevance of PGx and cost	[16]
U-PGx (Ubiquitous Pharmacogenomics)	Implement PGx through a pre-emptive panel strategy; studies of the impact on patient outcomes and cost-effectiveness; exploratory analysis to understand PGx	[17]

Since the objectives and implementation strategies of these programs have been thoroughly summarized elsewhere [2, 17–19], this review sets the focus on some of the challenges these programs have encountered and covers the solutions that have been made for overcoming some of these barriers for the implementation of PGx in the clinic. Further, this review aims to provide convincing evidence of the several benefits of preemptive PGx testing that have been reported thus far.

### Evidence of cost-effectiveness

One of the major barriers to implementing pharmacogenomics in the clinic has been the amount of evidence showing testing effectiveness or cost-effectiveness on the clinical outcome, which would demonstrate the necessity of the testing. For broader implementation of PGx, it is essential to demonstrate the value and cost-effectiveness of testing to key decision-makers [24]. With major initiatives of PGx implementation and separately conducted clinical trials, the number of studies evaluating the benefit of preemptive PGx is growing rapidly (Table 2).

**Table 2:** Benefit of pharmacogenetic testing on clinical outcome

Study	Findings	Benefit	References
2019, Seven of University of Florida Health primary care clinics, 375 enrolled patients	Within the same subgroup of IM/PMs prescribed tramadol or codeine at baseline, *CYP2D6*-guided group experienced a 30% reduction in composite pain intensity compared with the usual care group.	Improved efficacy	[25]
2019, Meta-analysis of 5 randomized controlled trials (RCT), 1737 participants across five RCTs	Pharmacogenetic-guided therapy 1.71 times more likely to achieve symptoms remission relative to individuals who received usual treatment.	Improved efficacy	[26]
2018, 17 hospitals in the Netherlands, 1103 evaluable patients	Genotype-guided dosing compared with historical cohort reduced the relative risk of severe toxicity for DPYD*2A carriers, was safe in the single c.1679 T > G carrier, and decreased the toxicity risk in c.2846A > T carriers, although the risk was still higher for c.2846A > T carriers than wild-type patients.	Improved safety	[27]
2017, The randomized clinical Genetic Informatics Trial (GIFT), 1650 randomized patients	The numbers of individual events in the genotype-guided group vs the clinically guided group were 2 vs 8 for major bleeding (RR, 0.24; 95% CI, 0.05–1.15), 56 vs 77 for INR of 4 or greater (RR, 0.71; 95% CI, 0.51–0.99), and 33 vs 38 for venous thromboembolism (RR, 0.85; 95% CI, 0.54–1.34). Genotype-guided warfarin dosing, compared with clinically guided dosing, reduced the combined risk of major bleeding.	Improved safety	[28]
2016, AltheaDx, San Diego	Applying PGx guided recommendations across the patient population resulted in the elimination and/or replacement of one to three drugs and an estimated annual saving of US$621 per patient.	Reduced cost	[29]
2016, Netherlands Cancer Institute, Slotervaart Hospital and Canisius Wilhelmina Hospital, 2038 patients	The risk of fluoropyrimidine-induced toxicity was significantly reduced from 73% (95% CI, 58–85%) in historical controls (*n* = 48) to 28% (95% CI, 10–53%) by genotype-guided dosing (*P* < .001); drug-induced death was reduced from 10% to 0%. Total treatment cost per patient was lower for screening (€2772 [$3767]) than for non-screening (€2817 [$3828]).	Improved safety, reduced cost	[30]
2015,2015, The Department of Neurology, University Hospital Center Zagreb, 206 patients	Of patients in the genotype-guided group (*CYP2C9, VKORC*1), 97% did not have any major complications compared with the control group. Estimated total cost per patient had a nonsignificant difference between genotype-guided and control group. However, the mean cost of bleeding was estimated to have significant difference at €119.32 (95% CI: €41.95–202.69) in favor of the PGx group.	Improved safety, reduced cost	[31]
2015, AssureRx Health, Mayo Clinic, 258 patients	Gene-guided treatment raised the odds of clinical response by 2.3-fold, the guided group had a 53% greater improvement in depressive symptoms.	Improved efficacy	[32]
2015, College of Pharmacy, University of Utah, 1025 patients	Pre-emptive screening with a panel-based approach resulted in a significant reduction in hospitalizations (9.8% vs 16.1%, *P* = 0.027) and patient visits to the emergency department (4.4% vs 15.4%, *P* = 0.0002).	Reduced hospitalization, reduced cost	[33]
2015, Assurex Health, Mason, Prospectively generated cohort, Initially 2168 cases and 10,880 controls	Patients receiving PGx testing saved $1035.60 in total medication costs over 1 year compared to the usual care cohort (*P* = 0.007). PGx testing improved adherence compared to standard of care.	Reduced cost, improved adherence	[34]
2014, Vanderbilt University, PREDICT study, 10,000 patients	Comparison of pre-emptive testing and reactive genotyping revealed that 14,656 tests would have been generated with point of care genotyping—the pre-emptive approach saves genotyping test costs by reducing the number of ordered tests by 60%.	Reduced cost	[21]
2013, The EU-PACT trial, 455 patients	In the genotype-guided group, the mean percentage of time in therapeutic range was 7.0 percentage points higher than in the control group. Significantly lower incidence of excessive anticoagulation was detected in the genotype-guided group than in the control group. Fewer adjustments in the dose of warfarin were made in the genotype-guided group than in the control group.	Improved efficacy, improved safety	[35]
2012, Vanderbilt University Medical Center, 52,942 patients	Within a 5-year window, 64.8% of individuals were exposed to at least one medication with a PGx association. Three hundred eighty-three adverse events (95% CI, 212–552) among 52,942 individuals could be prevented with an effective preemptive genotyping program.	Improved safety	[36]
2012, Mayo Clinic, 44 patients	On average, a 7.2% reduction in depressive symptoms for study subjects in the unguided treatment group was detected, compared with a 31.2% reduction in overall score for subjects in the guided group (*P* = 0.002).	Improved safety	[37]
2010, Medco Health Solutions, Mayo Clinic, 3584 patients	*CYP2C9* and *VKORC1* genotyping of warfarin recipients resulted in 31% fewer hospitalizations overall and a 43% lower risk of hospitalization for bleeding or thromboembolism.	Reduced hospitalization, reduced cost	[38]

Evaluations of the cost-effectiveness of PGx are mostly limited to single gene-drug pairs, and the amount of information on the cost-effectiveness of multiplexed preemptive strategies is limited [39, 40]. The PREDICT study brought attention to the benefit of panel-based testing over single gene testing—the ordering of 14,656 genetic tests was avoided when data on multiple genes was available beforehand [21], thereby saving genotyping test costs by reducing the number of single tests by 60%. Cost-effectiveness has been addressed by studies outside of the major implementation initiatives as well. One study showed that patients who received PGx testing saved €916.77 ($1035.60) in total on medication costs over 1 year compared to the cohort of standard care [34]. A study conducted in the Netherlands estimated that the total cost per patient was lower when screening, resulting in a cost savings of €45 ($61) per patient [30]. In warfarin treatment, the incremental cost-effectiveness ratio of PGx-guided therapy was estimated to be €31,225 per quality-adjusted life-years compared to the control group [31]. A study by AltheaDx, which aimed to survey the benefits of pharmacogenetics on the medical management of patients, found an estimated annual saving of €549 ($621) per patient that was tested [29]. Since the overall costs of panel-based and single gene tests are similar, it is not surprising that multi-gene tests are more cost-effective with the additional benefit of genotypes being available at the time of medication order [21]. In a review of 44 economic evaluations of pharmacogenetics, 30% were found to be cost-effective and 27% even cost-saving, hence making it a realistic future prospect [41]. A study that modeled the economic impact of PGx guided treatment for depression estimated savings of €3504 ($3962) annually per patient even when the cost of testing was assumed to be €1760 ($2000) [42]. The cost of broad genetic testing is decreasing rapidly, and when considering microarrays for PGx, the cost for reports can be even lower.

### Clinical validity

Starting from September 2010, more than 10,000 patients have undergone preemptive, panel-based testing through the Vanderbilt Pharmacogenomic program [21]. The studies of the first 9589 individuals show that 91% of the genotyped patients had more than one actionable PGx variant. Further, the PG4KDS study identified that approximately 98.5% of whites and 99.1% of blacks in the US have at least one high-risk diplotype [2]. Similar results were obtained both by the Mayo RIGHT and eMERGE-PGx programs, showing that 99% and > 96% of samples, respectively, carry high priority PGx actionable variants [39, 43]. A study of the genotype data of 44,000 participants in the Estonian biobank reported that 99.8% of all the assessed individuals had a genotype associated with increased risks to at least one medication [44].

When considering the impact on efficacy, the improvement in clinical response and treatment outcome has been reported in several studies. The Mayo Clinic showed that treating depression guided by pharmacogenomic testing reduced depressive symptoms four times compared with the unguided group (31.2% vs 7.2% of reduction) [37]. Another study reported a 53% improvement in depressive symptoms in the guided group and 2.3 times the odds of a better clinical response [32]. A recent study conducted by the University of Florida revealed efficacy improvement among *CYP2D6* intermediate and poor metabolizers, where 24% of *CYP2D6*-guided participants reported more than a 30% reduction in pain intensity versus 0% of patients on usual care [25].

**Fig. 2:**
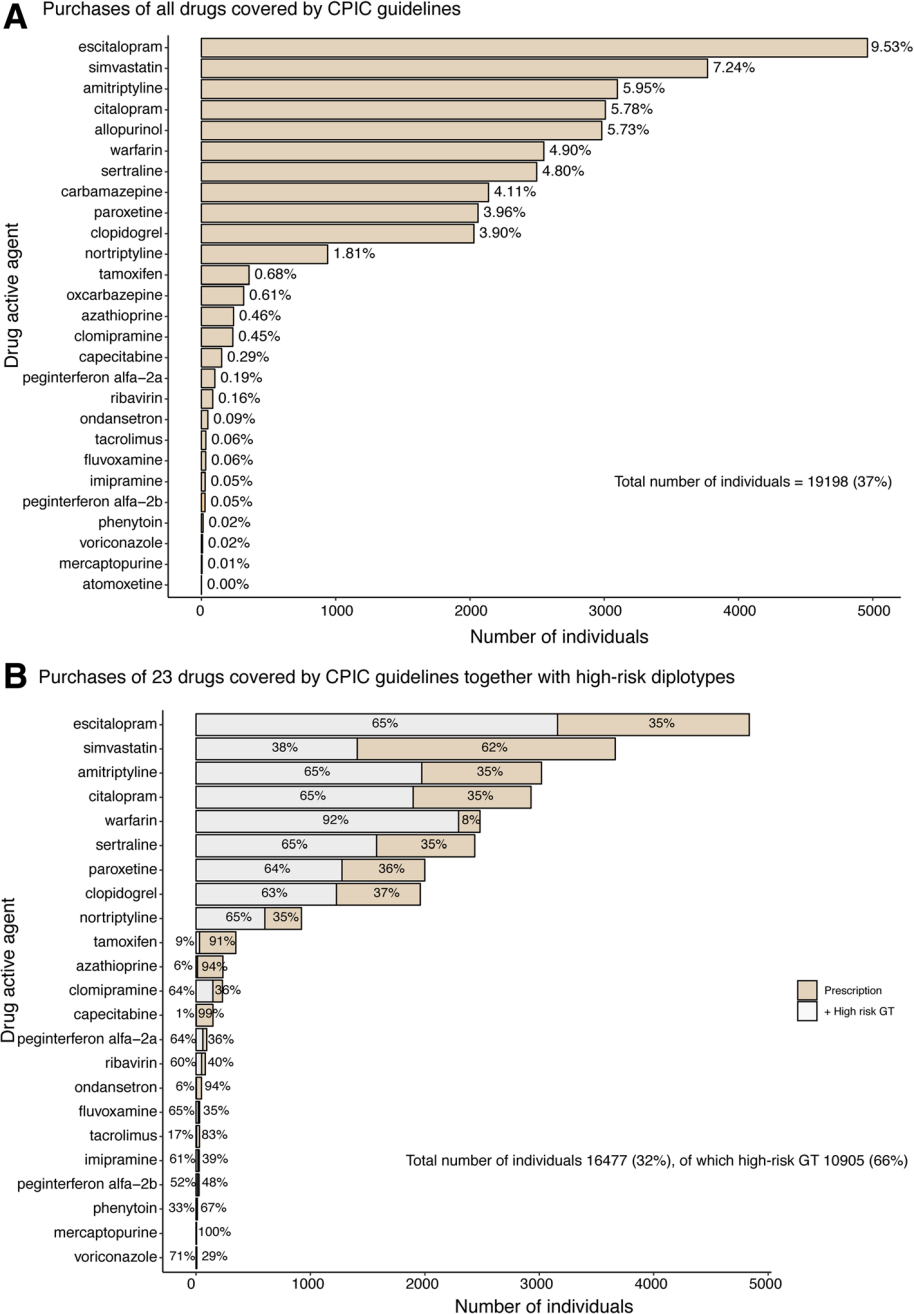
Purchasing of drugs with CPIC guidelines based on the Electronic health records of 52,000 Estonian biobank participants. **a** The number of individuals who have purchased at least one drug listed in CPIC guidelines. Percentages are indicating the proportions from the total number of biobank participants (52,062). **b** The number of individuals with wild-type or normal function genotypes and drug purchases (light gold), and the proportion of individuals with high-risk genotypes (gray) of a gene covered by the CPIC guidelines. Numbers are represented for 23 drugs since the pipeline for calling metabolizing phenotypes was developed for 11 genes [44]

One misconception of PGx testing is that it is only (or mostly) relevant for rare expensive drugs used in cancer treatment. However, when analyzing the number of individuals taking a pharmacogenomic-guided medication, an important study conducted at Vanderbilt found that 65% of the 52,000 surveyed individuals actually had been exposed to PGx medications [36]. Another study of the insurance claims of > 55 million individuals in the US reported that up to one quarter of patients had received a drug with a PGx recommendation label [45]. According to a study at St. Jude Children’s Research Hospital, during a 1-year period, 48% (2023/4245) of pediatric patients received at least one high-risk PGx drug [2]. Further, in the US generally, medications with PGx recommendations comprise 18% of all prescriptions [1], and 30 of the most commonly prescribed medications account for 738 million of yearly prescriptions [2]. Based on the Annual Statistics of the Estonian Agency of Medicines, a study indicated that almost 5.5% (55 defined daily dose (DDD)/1000 inhabitants/day) of individuals in the population use at least one of the studied PGx drugs on a daily basis, while in the Nordic countries, this proportion was even higher, 11.5–15.8% [44]. When analyzing the purchasing frequency of 46 PGx drugs, active agents listed at the CPIC guidelines (accessed 7 March 2019) based on the electronic health records of 52,000 participants at the Estonian biobank, we see that 37% (19,198/52062) of individuals have already received at least one prescription for the high-risk PGx drugs (Fig. 2a). When further analyzing the metabolizing phenotype predictions of 11 genes according to CPIC guidelines together with the drug purchasing data of 16,477 individuals, we see that 10,905 individuals with high-risk genotypes have been prescribed a corresponding medication (Fig. 2b). Thus, up to 66% (10,905/16,477) of prescriptions for individuals would need adjustment if recommendations accounted for high-risk genotypes.

Finally, probably the most important factor highlighting the necessity of PGx testing is the possibility of avoiding adverse drug events (ADE). A study conducted in the Netherlands revealed that preemptive *DPYD* genotyping and guided dosing reduced the risk of fluoropyrimidine-induced toxicity in historical controls from 73 to 28% and the number of drug-induced deaths was reduced from 10 to 0% [30]. A study done at the Mayo Clinic reported that, compared with the control group, *CYP2C9* and *VKORC1* genotyping resulted in a 43% lower risk of hospitalization for bleeding or thromboembolism and 31% fewer hospitalizations overall [38]. Another study on warfarin found that genotype-guided treatment significantly reduced the combined risk for major bleeding [28]. Further, a Vanderbilt prediction study estimated that across six medication and ADE combinations among 52, 942 individuals, 383 of the adverse events could have been prevented with preemptive genotyping [36].

It should be acknowledged that all of these studies have indicated that the amount of individuals who would benefit from effective preemptive testing is tremendous and that clear evidence of the necessity of testing is present.

### Acceptance of PGx testing

PGx implementation is highly dependent on its general acceptance among patients and healthcare professionals, which is probably one of the most influential prerequisites for effective and successful implementation. Among clinicians, the main cause of resistance to widespread implementation appears to be unfamiliarity with PGx data or lack of genetics knowledge. Healthcare providers who completed their training more than 10 years ago probably had little to no genomic medicine in their programs. Furthermore, technology and discoveries in genomics have advanced at tremendous speed, making it very difficult to stay updated on all the novel opportunities. Although the scientific evidence and clinical benefit of PGx is strong, it can all remain unclear due to poor literacy in genomics, which lowers the overall acceptance. This was an obstacle all acknowledged with the launch of the first PGx initiatives, which led to better solutions, starting with the increased availability of pharmacogenomic educational materials and programs.

Surveys, which have been conducted for assessing the general situation among healthcare providers, have shown overall acceptance of the need for PGx testing. The results of different surveys show high percentages such as 97.6% [46], 99.7% [47], 99% [48], and 84.3% [49] of healthcare professionals who believe in the concept of pharmacogenomics or find it relevant for clinical practice. However, when asked about the level of knowledge and readiness for interpretation of testing results, only 10.3% [46], 14.1% [47], and 13% [50] felt adequately informed about pharmacogenomic testing, and 88.8% [47] to 96.6% [51] said they would like to receive additional training on PGx. These surveys show that the overall acceptance of PGx implementation is high, but further time should be dedicated to provide more educational materials and courses. This is also supported by a survey done among prescribers who had attended educational courses, which showed that healthcare professionals felt adequately informed to use PGx results in their clinical practice [48].

**Fig. 3:**
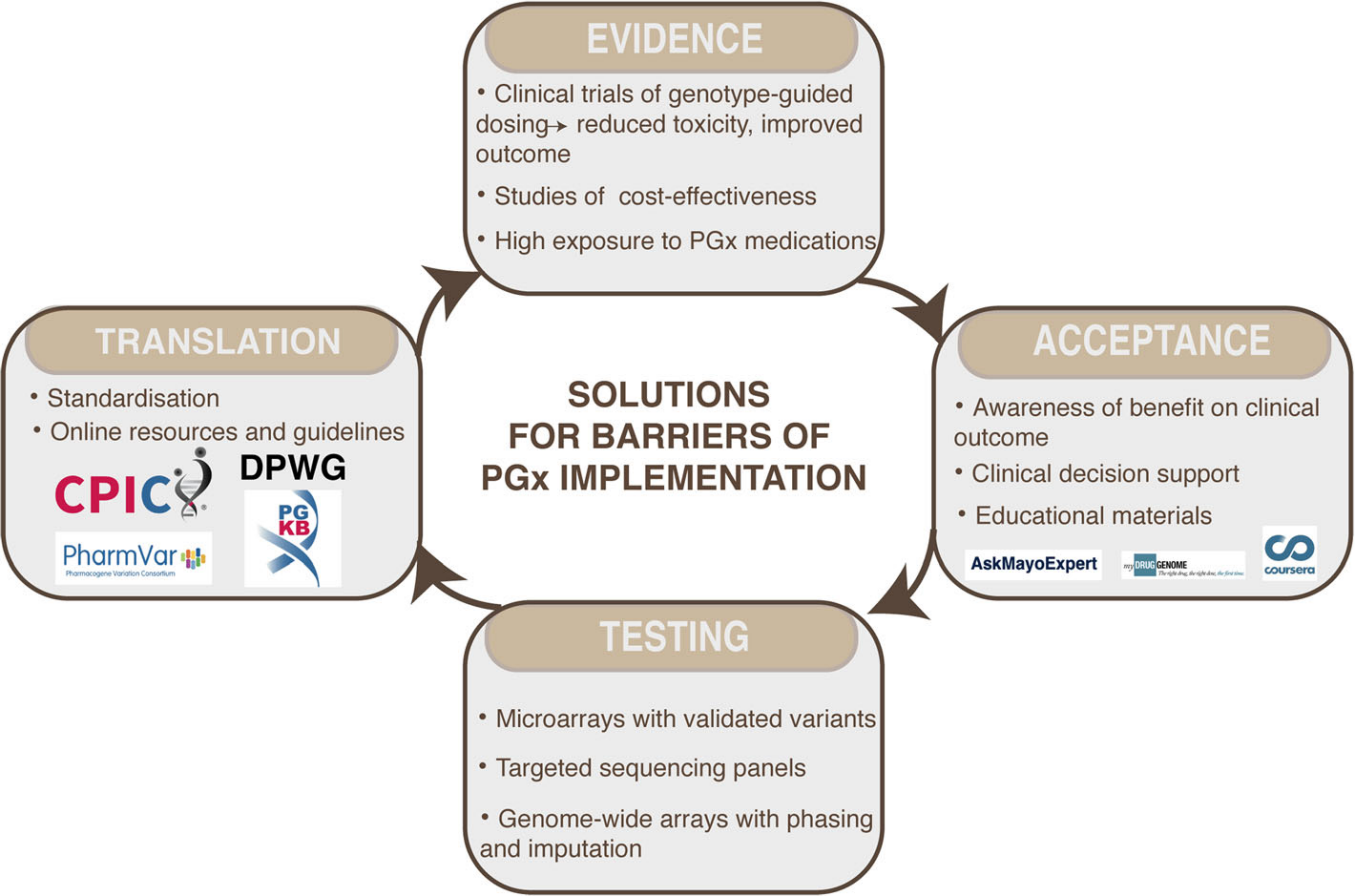
Current solutions and opportunities for overcoming some of the barriers of pharmacogenetic implementation

For now, several resources have been developed by different PGx implementation initiatives to raise the competence of clinicians in PGx (Fig. 3). PharmGKB provides a tab with resources that contains a collection of links to educational materials. Furthermore, Vanderbilt University developed the “My Drug Genome” portal (www.mydruggenome.org), for learning how genetics affect drug response. They also supported the development of a Coursera online Course in Personalized Medicine (www.coursera.org/learn/personalizedmed/). The Mayo Clinic has created numerous educational materials (“AskMayoExpert”, online videos/modules) directed to both clinicians and patients with the goal of enhancing general knowledge and implementation [52]. St. Jude Children’s Research Hospital enables tracking of implemented gene/drugs on a website together with implementation-specific publications and presentations [22]. U-PGx has developed an e-learning platform for distributing general PGx knowledge suitable for physicians and pharmacists [17].

### Automated decision support tools for PGx integration

One effective tool that helps clinicians with limited knowledge, and an essential component for the smooth implementation of PGx, is the availability of clinical decision support software (CDS). The opportunity to activate a CDS at the time of ordering a high-risk drug is a vital factor when preemptively testing. All ongoing initiatives are dedicated to resolving the issue of technical resources needed for PGx-guided treatment and several CDS designs have already been launched [53]. Several strategies are available in the form of active vs passive and pre- vs post-test alerts. When PGx information is not preemptively available, pre-test alerts are used to motivate clinicians to first order a genotype test before prescribing a drug [54]. Common across the implementation studies is the use of electronic health records to facilitate the delivery of CDS as an active alert at the time of prescribing or passively as part of the digital records [53, 55]. It is necessary to have guiding PGx results available for clinicians at any time through passive CDS in the form of reports of relevant PGx recommendations [2].

CDS systems can be used at the time of prescribing a high-risk drug and provide automated recommendations indicating why certain modifications should be applied to the selected drug or dose.

In a study of the impact of the availability of preemptive pharmacogenomic genotyping results, an institutional clinical decision support system provided pharmacogenomic recommendations using traffic light alerts. As physicians had modest knowledge and minimal to no prior experience with using pharmacogenomics, the goal was to minimize complexity by designing a CDS that enabled clinicians to understand the implications of the recommendations without necessarily knowing genomics. The results supported this approach—medications with high pharmacogenomic risk were changed and no high-risk drugs were prescribed during the entire study [56]. In the case of preemptive testing, when a patient’s test results are already in place in the EHRs, before the prescription of a high-risk drug, a system to actively deliver the patient’s drug-specific information based on existing genetic test results is essential [2]. In countries with digital health and prescription information systems, CDS systems have the potential to help increase the widespread acceptance and knowledge required for the implementation of PGx into clinical settings.

The U-PGx PREPARE study also developed solutions for sites with limited EHR infrastructure. The “Safety-Code” card is part of a mobile CDS, and with a quick response code, a medical professional is directed to a website with dosing recommendations customized for the patient [55]. In addition, an overview of the most relevant PGx test results with a list of drugs that have PGx guided recommendations is also listed on the card.

### A platform for PGx testing

Ongoing implementation studies are currently applying different sequencing or microarray-based genotyping technologies for preemptive PGx testing. The major question to tackle is which variants or genes to test and how to test them. Several solutions have been established (Fig. 3), but some give rise to new challenges to overcome. Commercial and ready-to-use, targeted genotyping assays probe for preselected variants with well-defined associations and recommendations, and usually, a selection of common variants across specific genes is screened for. One of the first PGx arrays was the Drug Metabolizing Enzymes and Transporters (DMET) Plus array by Affymetrix (now Thermo Fisher Scientific), which enables the simultaneous analysis of 1936 SNPs and 5 CNVs in 231 pharmacogenes [57]. This array is used, for example, for PGx implementation in the two PGx initiatives: the 1200 Patients project by the University of Chicago [16] and the PG4KDS protocol at St. Jude Children’s Research Hospital [11]. The initial platform for the PREDICT study was Illumina’s VeraCode ADME core panel, which tests 184 variants in 34 pharmacogenes [13]. The U-PGx PREPARE study is covering a panel of 50 variants in 13 pharmacogenes selected systematically by prespecified criteria [17]. There have been discussions of using a more comprehensive approach to define pharmacogenetic variation. Genotyping a selection of relevant PGx variants will miss newly identified but potentially clinically relevant alleles. For capturing these variants as well, arrays need to be renewed or complemented with a customized SNP assay. Another problem, in the case of genotyping arrays, is the different designs of assays that might make it difficult to compare the results from several genotyping platforms [58]. A study where a comparison of different genotyping systems was performed showed inconsistent haplotype calls for the same alleles because of differences in test designs [59]. There may also be discrepancies due to the assessment of copy number variants which, for example, in the case of *CYP2D6*, may lead to falsely identified metabolizer phenotypes [58].

With the rapid advancement in technology and the decrease in sequencing costs, a comprehensive option solving the aforementioned downsides of array-based testing would be to use genome sequencing for preemptive testing. However, we need to acknowledge the various barriers that need to be overcome in this area as well. Several recent studies have shown that more than 90% of the variants in pharmacogenes are rare [60, 61]. On the one hand, genotyping a selection of relevant PGx variants will miss novel but potentially clinically relevant alleles, but on the other hand, novel variants need to pass through functional validation studies before clinical implementation. When such variants can be detected without too much additional effort or cost, at least gathering the information for research purposes is highly valuable. The role of these rare variants in variable drug response will be more difficult to determine [39], as statistical methods typically used for common variants or overexpression studies for candidate validation are not feasible. Computational prediction methods can help us along the way when assessing the functional relevance of novel variants [62], but most of the computational prediction methods base their functional assessment on algorithms that are not adjusted for pharmacogenetic variants as they are calibrated on disease data sets [63]. The recent optimized prediction framework developed especially for pharmacogenetic assessments addressed this issue and designed a method that outperforms the previous computational algorithms [64]. Furthermore, aside from computational methods, the past decade has brought significant advancements in genome-editing with the bacterial clustered, regularly interspaced, short palindromic repeats (CRISPR)–Cas9 system, which has opened up comprehensive possibilities for experimental validation of novel variants [65, 66]. As these methods open up new possibilities for previous test results to change over time, for example, a wild-type allele may be reclassified into an allele with reduced or increased function, CDS tools that are developed need to include mechanisms for alerting clinicians when changes occur.

Although the costs associated with whole-genome sequencing continue to decline, they remain prohibitively expensive for wide clinical use, and the issue of storing large amounts of data can become a barrier as well. One great possibility is to use capture libraries for targeted sequencing of genes of interest in order to find a favorable balance between cost, throughput, and deep coverage [67]. This kind of approach is applied by the eMERGE initiative where targeted sequencing is applied to capture variation in 84 pharmacogenes called the PGRN-Seq panel [68]. When considering the best balance between cost and comprehensiveness, this approach currently seems like a very promising solution. For the rare variants, one of the objectives of eMERGE is to establish a repository of pharmacogenetic variants of unknown significance that are also linked to a repository of clinical phenotypes [68]. This information can be used for further pharmacogenomic discovery since sequence variants determined by PGRNseq will be available to the public through SPHINX (Sequence, Phenotype, and pHarmacogenomics INtegration eXchange, http://emergesphinx.org).

Another method for finding a balance between comprehensiveness and cost would be to use genome-wide genotyping arrays. Combining genotyping with phasing and imputation enables very similar comprehensive predictions of pharmacogenetically relevant alleles to be made, comparable to results obtained by genome sequencing [44]. Further, performing phasing also allows for more precise haplotype calls (see the “Translation into pharmacogenetic reports” section). Nevertheless, the challenges remain on the part of computational requirements and pipelines for performing imputation and assessing its accuracy; achieving high imputation accuracy requires population-specific reference panels for imputation [69]. In settings where this can be achieved, using genome-wide microarrays combined with imputed variants would be a highly cost-effective tool to pinpoint individuals who need altered dosing recommendations.

Technology will continue to develop and provide cheaper and more comprehensive approaches for preemptive pharmacogenomic testing. The current initiatives are all providing tremendous value. Initiatives that have taken a broader approach help to take pharmacogenetic discoveries further by expanding the list of variants that are functionally validated and with known significance. For now, both broad initiatives and programs that only cover validated variants advance our knowledge on the effectiveness and improved outcomes of pharmacogenetic testing.

### Translation into pharmacogenetic reports

With the start of the first pharmacogenetic implementation initiatives, several barriers emerged for the translation of PGx test results into clinical action. Admittedly, with that, several lessons were learned and opportunities for overcoming some of these barriers began to unfold. Currently, there are several resources available to support the translation of obtained information of pharmacogenetic genotypes into treatment recommendations (Fig. 3).

One of the first challenges, alongside the different platform choices for retrieving genotypes, was how to convert the results of a genetic test into clinical action. Anticipating this necessity for precise guidelines, were two consortia, the Dutch Pharmacogenetics Working Group (DPWG) [70, 71] and the Clinical Pharmacogenetics Implementation Consortium (CPIC) [72], who have now provided well-known therapeutic recommendations to facilitate the translation of pharmacogenetics. The guidelines of both groups instruct clinicians on dosing recommendations or alternative medication options for those carefully selected gene-drug pairs that have evidence-based, significant impacts on the outcome of pharmacotherapy, thus also helping to solve the question of which pharmacogenes that are relevant for testing. A comparison of these guidelines on the same gene–drug showed substantial similarities and observed discordances can be mostly explained by the use of different methodologies for dosing [73]. With ongoing collaboration, all of these differences discovered between the guidelines are being further addressed for standardization. As guidelines continue to evolve and expand, it is important to develop methods for keeping the information up to date when new content becomes available. This can pose a technical challenge for developing a system that regularly updates available guidelines.

Having genotype data and guidelines with the therapeutic recommendations of gene-drug pairs available, one of the next important questions and challenges is how to translate the genotype data at hand into phenotype information. Curated databases such as CPIC [74] together with Pharmacogenomics Knowledge Base (PharmGKB) [75, 76] are now offering translation tables on how to define pharmacogenetic alleles on the basis of genetic variation and, furthermore, how to assign diplotypes to interpreted phenotypes. However, diplotype assignment still remains somewhat challenging based on both microarray and sequencing data. It is currently not straightforward to optimally translate individual-level genotype data into diplotypes and further to associated phenotypes based on the tables offered. Actionable alleles in tables that contain multiple variants make diplotype assignment one of the first challenges. Short reads and genotyping data are often unable to resolve haplotype information, thus, simultaneous reading of both parental alleles makes it difficult to determine the correct phase. One of the solutions for identifying variants co-located on the same chromosome is computational phasing and several well-known algorithms have been designed for that [77, 78]. However, in the case of the most important family of pharmacogenes—the Cytochrome P450s—it is known they are very polymorphic and exhibit sequence similarities of between 71 and 80% [79]. The CYP2D6 enzyme, metabolizing around 25% of the commonly prescribed drugs, harbors more than 150 known allelic variations [80], deletions and duplications, structural rearrangements, and repetitive elements, thus making short-read sequencing and phasing challenging [81]. The complete solution would be long-read sequencing technologies, sufficient to span the distance between markers of interest [78, 81]. However, due to current costs, long-read sequencing platforms are not widely used and since they are not yet suitable for concurrent sequencing of multigene panels, in the case of pharmacogenetic genotyping, they act more as an addition to short-read sequencing than an alternative [63].

Another possibility for resolving haplotype information was introduced by PharmCAT, the Pharmacogenomics Clinical Annotation Tool. The idea was to first give a score to an allele based on the number of variant positions used to define the allele, then permutate possible combinations of sample genotypes and attempt to match each to an allele, finally only returning the top-scoring diplotype [82]. The goal of PharmCAT is to develop a software tool to standardize diplotype assignments based on the allele definitions from genetic variants and enable this regardless of where the genetic test is being performed [83]. Standardization is one of the remaining barriers for consistent and effective implementation of pharmacogenomics, and efforts like PharmCAT are underway to address this issue [84].

One of the remaining major challenges in implementing both sequencing and genotyping data is the confusion surrounding the nomenclature for reporting the variants tested and used to match diplotypes to phenotypes. The most commonly used nomenclature in pharmacogenomics, which is currently also the basis of translation tables, is the star (*) allele nomenclature system, which describes haplotype patterns defined at the gene level. The *1 allele is usually the most common allele in all populations, a reference sequence that codes a functional protein product and all other numeric labels define haplotypes carrying one or more alternative variants [85]. The reference allele is often assigned in the absence of variants defining other alleles, thus a *1 designation depends on the variants interrogated. Reporting only star alleles makes it difficult to determine the variants studied; therefore, to interpret genetic test results, knowledge of all the variants tested is necessary [58].

However, first and foremost when reporting PGx, a standardization of tested variants should be done. A comparison of the results of PGx testing from different laboratories, a study conducted by Centers for Disease Control and the Prevention-based Genetic Testing Reference Material Coordination Program, revealed many inconsistencies due to different nomenclature systems and PGx test design [86]. Laboratories interrogated different sets of variants and this led to different haplotype calls for the same allele. When the results are implemented in the EHR, ambiguous results may follow a patient for a lifetime. Thus, variants that need to be tested for star allele designation should meet a minimum standard. There are currently efforts underway to address the issues with allele nomenclature. The Pharmacogene Variation Consortium (PharmVar) now expands its focus beyond the Human Cytochrome P450 Alleles by including other clinically important pharmacogenes, aiming to improve pharmacogenomics nomenclature by providing a repository of standardized variation data [87]. PharmVar offers several downloadable options displaying allelic data consistently across genes and shows coordinates of variants across all reference genome builds, while also listing haplotypes on which the variants can be found. Furthermore, the functional information is presented for all of the alleles, cross-referenced with PharmGKB, providing additional evidence levels for each haplotype, which can be especially relevant in the case of clinical implementation.

CPIC and Dutch guidelines, together with translation tables, offer thoroughly curated, evidence-based guidance for pharmacogenetic implementation. Straightforward instructions for adapting the guidelines are a significant milestone in the worldwide standardization of the implementation of pharmacogenetics.

## Conclusion

Surveys have reported that high percentages of healthcare professionals believe in the concept of pharmacogenomics or find it relevant in clinical practice. Admittedly, further time should be dedicated to training and educational activities to help clinicians feel more comfortable interpreting the results and raise their overall competence in the field. Current implementation programs are already making more training opportunities available. Further, consortia like the CPIC have provided guidelines to make genetic results easier to implement and interpret, and when these are accompanied by automated decision support software for clinicians, introductory training should be sufficient for clinicians. Research studies have identified relevant pharmacogenetic variants that can already be used for implementation to change the way drugs are prescribed. For the systematic implementation of preemptive PGx, more standardization of interrogated variants is necessary between different initiatives. One solution for the consistent translation of variants into metabolizing phenotypes can be achieved by setting a minimum standard for variants required to be tested for the designation of alleles as well as having straightforward instructions for using the translation tables. Databases like PharmVar are focusing on addressing nomenclature standardization. Economic and efficacy evaluations have provided evidence of the vast benefit of genotype-guided treatment and more studies of using PGx are well underway. All these ongoing initiatives have turned several challenges in PGx implementation into solutions, thus making the promise of pharmacogenomics a reality.

As a future direction, biobanks can be regarded as untapped resources for both identifying rare variants and for validation studies. They can also be used for studying challenges and solutions of PGx implementation in general. The existing broad and longitudinal data on biobank participants can be used for translation of genotype data of pharmacogenes into recommendations for more improved and more cost-effective drug treatment. In addition, providing feedback on the relevant PGx information back to biobank participants enables further studies to evaluate the benefit of preemptive PGx testing, thus illustrating the potential role of biobanks in PGx implementation. As research continues, the evidence of gene–drug associations will increase and implementation barriers faced today will be resolved. In the very near future, it will not be unusual for patients to have their PGx information available for improved treatment success and decreased societal costs. Although different methods have their limitations, we should not let the perfect become the enemy of the good and halt the implementation of what has currently been shown to improve treatment outcomes and reduce adverse events in a cost-effective manner.

## Data Availability

Not applicable for this review.

## Abbreviations

ADE: Adverse drug events
CDS: Clinical decision support
CPIC: Clinical Pharmacogenetics Implementation Consortium
CRISPR: Bacterial clustered regularly interspaced short palindromic repeats
DDD: Defined daily dose
DMET: Drug Metabolizing Enzymes and Transporters
DPWG: Dutch Pharmacogenetics Working Group
EHR: Electronic health records
eMERGE: electronic Medical Records and Genomics
GIFT: The randomized clinical Genetic Informatics Trial
PGRN: The Pharmacogenomics Research Network
PGx: Pharmacogenomics
PharmCAT: The Pharmacogenomics Clinical Annotation Tool
PharmGKB: Pharmacogenomics Knowledge Base
PharmVar: The Pharmacogene Variation Consortium
PREDICT: Pharmacogenomic Resource for Enhanced Decisions in Care and Treatment program
PREPARE: PREemptive Pharmacogenomic testing for prevention of Adverse drug REactions
RCT: Randomized controlled trials
SEAPharm: The South East Asian Pharmacogenomics Research Network
SPHINX: Sequence, Phenotype, and pHarmacogenomics INtegration eXchange
U-PGx: Ubiquitous Pharmacogenomics

## References

[1] Relling MV, Evans WE (2015). Pharmacogenomics in the clinic. Nature.

[2] Dunnenberger HM (2015). Preemptive clinical pharmacogenetics implementation: currentprograms in five United States medical centers. Annu Rev Pharmacol Toxicol.

[3] The African American Cardiovascular Pharmacogenomics Consortium. [Online]. Available: https://precisionmedicine4all.com.

[4] Gottesman O (2013). The CLIPMERGE PGx program: clinical implementation of personalized medicine through electronic health records and genomics-pharmacogenomics. Clin Pharmacol Ther.

[5] Gottesman O (2013). The electronic medical records and genomics (eMERGE) network: past, present, and future. Genet Med.

[6] Genomic and outcomes database for pharmacogenomics and implementation studies (Go-PGx). [Online]. Available: https://www.genomecanada.ca/en/genomic-and-outcomes-database-pharmacogenomics-and-implementation-studies-go-pgx.

[7] Weitzel KW (2016). The IGNITE network: a model for genomic medicine implementation and research. BMC Med Genet.

[8] Eadon MT (2016). Implementation of a pharmacogenomics consult service to support the INGENIOUS trial. Clin Pharmacol Ther.

[9] Teng K (2014). Institutional profile: Cleveland clinic’s center for personalized healthcare: setting the stage for value-based care. Pharmacogenomics.

[10] Johnson JA (2013). Institutional Profile Medinice Programm: clinical implementation of pharmacogenetics. Pharmacogenomics.

[11] Hoffman JM (2014). PG4KDS: a model for the clinical implementation of pre-emptive pharmacogenetics. Am J Med Genet C Semin Med Genet.

[12] Shuldiner AR (2013). The pharmacogenomics research network translational pharmacogenetics program: overcoming challenges of real-world implementation. Clin Pharmacol Ther.

[13] Pulley JM (2012). Operational implementation of prospective genotyping for personalized medicine: the design of the vanderbilt PREDICT project. Clin Pharmacol Ther.

[14] Wang L (2014). Preemptive genotyping for personalized medicine: design of the right drug, right dose, right time – using genomic data to individualize treatment protocol. Mayo Clin Proc.

[15] South East Asian Pharmacogenomics Research Networ (SEAPHARM). [Online]. Available: https://www.ims.riken.jp/english/projects/pj09.php.

[16] O’Donnell PH (2012). The 1200 patients project: creating a new medical model system for clinical implementation of pharmacogenomics. Clin Pharmacol Ther.

[17] van der Wouden CH (2017). Implementing pharmacogenomics in Europe: design and implementation strategy of the ubiquitous pharmacogenomics consortium. Clin Pharmacol Ther.

[18] Volpi S (2018). Research directions in the clinical implementation of pharmacogenomics: an overview of US programs and projects. Clin Pharmacol Ther.

[19] Klein Michelle E., Parvez Md Masud, Shin Jae-Gook (2017). Clinical Implementation of Pharmacogenomics for Personalized Precision Medicine: Barriers and Solutions. Journal of Pharmaceutical Sciences.

[20] Bush W (2016). Genetic variation among 82 pharmacogenes: the PGRNseq data from the eMERGE network. Clin Pharmacol Ther.

[21] Van Driest SL (2014). Clinically actionable genotypes among 10,000 patients with preemptive pharmacogenomic testing. Clin Pharmacol Ther.

[22] Luzum JA (2017). The pharmacogenomics research network translational pharmacogenetics program: outcomes and metrics of pharmacogenetic implementations across diverse healthcare systems. Clin Pharmacol Ther.

[23] Chan L, Sani LL, Quah CB. Pharmacogenomics pharmacogenomics in Asia : a systematic. Futur Med. 10(2217):2017, 2017–0009. 10.2217/pgs-2017-0009 28594321

[24] Patrinos GP, Mitropoulou C (2017). Measuring the value of pharmacogenomics evidence. Clin Pharmacol Ther.

[25] Smith DM, et al. CYP2D6-guided opioid therapy improves pain control in CYP2D6 intermediate and poor metabolizers: a pragmatic clinical trial. Genet Med. 2019;0(0). 10.1038/s41436-018-0431-8 PMC6650382 30670877

[26] Bousman CA, Arandjelovic K, Mancuso SG, Eyre HA, Dunlop BW (2019). Pharmacogenetic tests and depressive symptom remission: a meta-analysis of randomized controlled trials. Pharmacogenomics.

[27] Lunenburg CATC (2016). Prospective DPYD genotyping to reduce the risk of fluoropyrimidine-induced severe toxicity: ready for prime time. Eur J Cancer.

[28] Gage BF (2017). Effect of genotype-guided warfarin dosing on clinical events and anticoagulation control among patients undergoing hip or knee arthroplasty: the GIFT randomized clinical trial. JAMA.

[29] Saldivar JS (2016). Initial assessment of the benefits of implementing pharmacogenetics into the medical management of patients in a long-term care facility. Pharmgenomics Pers Med..

[30] Deenen MJ (2016). Upfront genotyping of DPYD^∗^2A to individualize fluoropyrimidine therapy: a safety and cost analysis. J Clin Oncol.

[31] Mitropoulou C (2015). Economic evaluation of pharmacogenomic-guided warfarin treatment for elderly Croatian atrial fibrillation patients with ischemic stroke. Pharmacogenomics.

[32] Altar CA, Carhart J, Allen JD, Hall-Flavin D, Winner J, Dechairo B (2015). Clinical utility of combinatorial pharmacogenomics-guided antidepressant therapy: evidence from three clinical studies. Mol Neuropsychiatry.

[33] Brixner D (2016). The effect of pharmacogenetic profiling with a clinical decision support tool on healthcare resource utilization and estimated costs in the elderly exposed to polypharmacy. J Med Econ.

[34] Winner JG (2015). Combinatorial pharmacogenomic guidance for psychiatric medications reduces overall pharmacy costs in a 1 year prospective evaluation. Curr Med Res Opin.

[35] Pirmohamed M (2013). A randomized trial of genotype-guided dosing of warfarin. N Engl J Med.

[36] Schildcrout J S, Denny J C, Bowton E, Gregg W, Pulley J M, Basford M A, Cowan J D, Xu H, Ramirez A H, Crawford D C, Ritchie M D, Peterson J F, Masys D R, Wilke R A, Roden D M (2012). Optimizing Drug Outcomes Through Pharmacogenetics: A Case for Preemptive Genotyping. Clinical Pharmacology & Therapeutics.

[37] Hall-Flavin DK (2012). Using a pharmacogenomic algorithm to guide the treatment of depression. Transl Psychiatry.

[38] Epstein RS (2010). Warfarin genotyping reduces hospitalization rates. Results from the MM-WES (Medco-Mayo Warfarin Effectiveness Study). J Am Coll Cardiol.

[39] Roden DM (2018). Benefit of preemptive pharmacogenetic information on clinical outcome. Clin Pharmacol Ther.

[40] Prescott WA, Doloresco F, Brown J, Paladino JA (2010). Cost effectiveness of pharmacogenomics: a critical and systematic review. Pharmacoeconomics.

[41] Verbelen M, Weale ME, Lewis CM (2017). Cost-effectiveness of pharmacogenetic-guided treatment: are we there yet?. Pharmacogenomics J.

[42] Maciel A, Cullors A, Lukowiak AA, Garces J (2018). Estimating cost savings of pharmacogenetic testing for depression in real-world clinical settings. Neuropsychiatr Dis Treat.

[43] Ji Y (2016). Preemptive pharmacogenomic testing for precision medicine: a comprehensive analysis of five actionable pharmacogenomic genes using next-generation DNA sequencing and a customized CYP2D6 genotyping cascade. J Mol Diagn.

[44] Reisberg Sulev, Krebs Kristi, Lepamets Maarja, Kals Mart, Mägi Reedik, Metsalu Kristjan, Lauschke Volker M., Vilo Jaak, Milani Lili (2018). Translating genotype data of 44,000 biobank participants into clinical pharmacogenetic recommendations: challenges and solutions. Genetics in Medicine.

[45] Frueh FW (2008). Pharmacogenomic biomarker information in drug labels approved by the United States Food and Drug Administration: prevalence of related drug use. Pharmacotherapy.

[46] Stanek EJ (2012). Adoption of pharmacogenomic testing by US physicians: results of a nationwide survey. Clin Pharmacol Ther.

[47] Bank PC, Swen JJ, Guchelaar HJ (2017). A nationwide survey of pharmacists’ perception of pharmacogenetics in the context of a clinical decision support system containing pharmacogenetics dosing recommendations. Pharmacogenomics.

[48] Peterson JF (2016). Attitudes of clinicians following large-scale pharmacogenomics implementation. Pharmacogenomics J.

[49] Just KS, Steffens M, Swen JJ, Patrinos GP, Guchelaar HJ, Stingl JC (2017). Medical education in pharmacogenomics—results from a survey on pharmacogenetic knowledge in healthcare professionals within the European pharmacogenomics clinical implementation project Ubiquitous Pharmacogenomics (U-PGx). Eur J Clin Pharmacol.

[50] Haga S, Burke W, Ginsburg G, Mills R, Agans R (2012). Primary care physicians’ knowledge of and experience with pharmacogenetic testing. Clin Genet.

[51] Robb L (2013). An evaluation of pharmacists’ expectations towards pharmacogenomics. Pharmacogenomics.

[52] Caraballo PJ (2017). Multidisciplinary model to implement pharmacogenomics at the point of care. Genet Med.

[53] Hinderer M (2017). Integrating clinical decision support systems for pharmacogenomic testing into clinical routine- a scoping review of designs of user-system interactions in recent system development. BMC Med Inform Decis Mak.

[54] Herr TM, Peterson JF, Rasmussen LV, Caraballo PJ, Peissig PL, Starren JB (2018). Pharmacogenomic clinical decision support design and multi-site process outcomes analysis in the eMERGE Network. J Am Med Informatics Assoc.

[55] Blagec Kathrin, Koopmann Rudolf, Crommentuijn – van Rhenen Mandy, Holsappel Inge, van der Wouden Cathelijne H, Konta Lidija, Xu Hong, Steinberger Daniela, Just Enrico, Swen Jesse J, Guchelaar Henk-Jan, Samwald Matthias (2018). Implementing pharmacogenomics decision support across seven European countries: The Ubiquitous Pharmacogenomics (U-PGx) project. Journal of the American Medical Informatics Association.

[56] O’Donnell PH (2017). Pharmacogenomics-based point-of-care clinical decision support significantly alters drug prescribing. Clin Pharmacol Ther.

[57] Arbitrio M, et al. DMET™ (Drug Metabolism Enzymes and Transporters): a pharmacogenomic platform for precision medicine. Oncotarget. 2016;7(33):54028–50. 10.18632/oncotarget.9927 PMC5288240 27304055

[58] Kalman LV (2016). Pharmacogenetic allele nomenclature: international workgroup recommendations for test result reporting. Clin Pharmacol Ther.

[59] Pratt VM (2010). Characterization of 107 genomic DNA reference materials for CYP2D6, CYP2C19, CYP2C9, VKORC1, and UGT1A1: a GeT-RM and association for molecular pathology collaborative project. J Mol Diagnostics.

[60] Ingelman-Sundberg M, Mkrtchian S, Zhou Y, Lauschke VM. Integrating rare genetic variants into pharmacogenetic drug response predictions. Hum Genomics. 2018;12(1):26. 10.1186/s40246-018-0157-3 PMC5968569 29793534

[61] Wright GEB, Carleton B, Hayden MR, Ross CJD (2018). The global spectrum of protein-coding pharmacogenomic diversity. Pharmacogenomics J.

[62] Hassan Marwa S., Shaalan A.A., Dessouky M.I., Abdelnaiem Abdelaziz E., ElHefnawi Mahmoud (2019). Evaluation of computational techniques for predicting non-synonymous single nucleotide variants pathogenicity. Genomics.

[63] Lauschke Volker M, Ingelman-Sundberg Magnus (2016). Requirements for comprehensive pharmacogenetic genotyping platforms. Pharmacogenomics.

[64] Zhou Y, Mkrtchian S, Kumondai M, Hiratsuka M, Lauschke VM (2019). An optimized prediction framework to assess the functional impact of pharmacogenetic variants. Pharmacogenomics J.

[65] Shalem O, Sanjana NE, Zhang F (2015). High-throughput functional genomics using CRISPR-Cas9. Nat Rev Genet.

[66] Adli M. The CRISPR tool kit for genome editing and beyond. Nat Commun. 2018;9(1):1911. 10.1038/s41467-018-04252-2 PMC5953931 29765029

[67] Gordon AS, Fulton RS, Qin X, Mardis ER, Nickerson DA, Scherer S (2016). PGRNseq: a targeted capture sequencing panel for pharmacogenetic research and implementation. Pharmacogenet Genomics.

[68] Rasmussen-Torvik LJ (2014). Design and anticipated outcomes of the eMERGE-PGx project: a multicenter pilot for preemptive pharmacogenomics in electronic health record systems. Clin Pharmacol Ther.

[69] Mitt M (2017). Improved imputation accuracy of rare and low-frequency variants using population-specific high-coverage WGS-based imputation reference panel. Eur J Hum Genet.

[70] Swen JJ (2018). Pharmacogenetic information in clinical guidelines: the European perspective. Clin Pharmacol Ther.

[71] Swen JJ (2011). Pharmacogenetics: from bench to byte--an update of guidelines. Clin Pharmacol Ther.

[72] Relling MV, Klein TE (2009). CPIC: clinical pharmacogenetics implementation consortium of the pharmacogenomics research network. Clin Pharmacol Ther.

[73] Bank PCD (2018). Comparison of the guidelines of the clinical pharmacogenetics implementation consortium and the Dutch pharmacogenetics working group. Clin Pharmacol Ther.

[74] Caudle K (2014). Incorporation of pharmacogenomics into routine clinical practice: the clinical pharmacogenetics implementation consortium (CPIC) guideline development process. Curr Drug Metab.

[75] Whirl-Carrillo M (2012). Pharmacogenomics knowledge for personalized medicine. Clin Pharmacol Ther.

[76] Barbarino Julia M., Whirl-Carrillo Michelle, Altman Russ B., Klein Teri E. (2018). PharmGKB: A worldwide resource for pharmacogenomic information. Wiley Interdisciplinary Reviews: Systems Biology and Medicine.

[77] Browning SR, Browning BL (2011). Haplotype phasing: existing methods and new developments. Nat Rev Genet.

[78] Choi Y, Chan AP, Kirkness E, Telenti A, Schork NJ (2018). Comparison of phasing strategies for whole human genomes. PLoS Genet.

[79] Lewis DFV, Watson E, Lake BG (1998). Evolution of the cytochrome P450 superfamily: sequence alignments and pharmacogenetics. Mutat Res.

[80] Zhou Y, Ingelman-Sundberg M, Lauschke VM (2017). Worldwide distribution of cytochrome P450 alleles: a meta-analysis of population-scale sequencing projects. Clin Pharmacol Ther.

[81] Yang Yao, Botton Mariana R, Scott Erick R, Scott Stuart A (2017). Sequencing the CYP2D6 gene: from variant allele discovery to clinical pharmacogenetic testing. Pharmacogenomics.

[82] NamedAlleleMatcher 101. [Online]. Available: https://github.com/PharmGKB/PharmCAT/wiki/NamedAlleleMatcher-101.

[83] Klein Teri E., Ritchie Marylyn D. (2017). PharmCAT: A Pharmacogenomics Clinical Annotation Tool. Clinical Pharmacology & Therapeutics.

[84] Caudle Kelly E, Keeling Nicholas J, Klein Teri E, Whirl-Carrillo Michelle, Pratt Victoria M, Hoffman James M (2018). Standardization can accelerate the adoption of pharmacogenomics: current status and the path forward. Pharmacogenomics.

[85] Robarge JD, Li L, Desta Z, Nguyen A, Flockhart DA (2007). The star-allele nomenclature: retooling for translational genomics. Clin Pharmacol Ther.

[86] Pratt VM (2016). Characterization of 137 genomic DNA reference materials for 28 pharmacogenetic genes: a GeT-RM collaborative project. J Mol Diagnostics.

[87] Gaedigk A, Sangkuhl K, Whirl-Carrillo M, Twist GP, Klein TE, Miller NA (2019). The evolution of PharmVar. Clin Pharmacol Ther.

